# Effects of visual map complexity on the attentional processing of landmarks

**DOI:** 10.1371/journal.pone.0229575

**Published:** 2020-03-02

**Authors:** Julian Keil, Dennis Edler, Lars Kuchinke, Frank Dickmann

**Affiliations:** 1 Geography Department, Cartography, Ruhr University Bochum, Bochum, Germany; 2 Methodology and Evaluation, International Psychoanalytic University Berlin, Berlin, Germany; Bruno Kessler Foundation, ITALY

## Abstract

In the era of smartphones, route-planning and navigation is supported by freely and globally available web mapping services, such as OpenStreetMap or Google Maps. These services provide digital maps, as well as route planning functions that visually highlight the suggested route in the map. Additionally, such digital maps contain landmark pictograms, i.e. representations of salient objects in the environment. These landmark representations are, amongst other reference points, relevant for orientation, route memory, and the formation of a cognitive map of the environment. The amount of visible landmarks in maps used for navigation and route planning depends on the width of the displayed margin areas around the route. The amount of further reference points is based on the visual complexity of the map. This raises the question how factors like the distance of landmark representations to the route and visual map complexity determine the relevance of specific landmarks for memorizing a route. In order to answer this question, two experiments that investigated the relation between eye fixation patterns on landmark representations, landmark positions, route memory and visual map complexity were carried out. The results indicate that the attentional processing of landmark representations gradually decreases with an increasing distance to the route, decision points and potential decision points. Furthermore, this relation was found to be affected by the visual complexity of the map. In maps with low visual complexity, landmark representations further away from the route are fixated. However, route memory was not found to be affected by visual complexity of the map. We argue that map users might require a certain amount of reference points to form spatial relations as a foundation for a mental representation of space. As maps with low visual complexity offer less reference points, people need to scan a wider area. Therefore, visual complexity of the area displayed in a map should be considered in navigation-oriented map design by increasing displayed margins around the route in maps with a low visual complexity. In order to verify our assumption that the amount of reference points not only affects visual attention processes, but also the formation of a mental representation of space, additional research is required.

## Introduction

Navigation is a complex everyday task that is executed when someone intends to reach a desired location. Independent of the mode and distance of travel, people need to compare their current position to their target position and choose a route that connects the two positions [1,2]. Following the chosen route requires them to continuously update their current position in order to identify the next required adjustment of the direction of travel [3].

In a familiar environment, people are able to plan a route and update their current position by resorting to their cognitive map, a previously learned mental representation of the environment [4–6]. However, in unfamiliar environments, they rely on external aids, such as maps or navigation systems [7].

Since the spread of smartphones, web mapping services like OpenStreetMap (OSM) and Google Maps are available almost everywhere. These services not only offer maps for free [8,9], they also provide route planning functions [10,11]. Additionally, landmarks, salient objects in the environment [12–14], are represented in these maps as pictograms. Landmark representations not only support the formation of a cognitive map, they also facilitate orientation, as users can match them to surrounding landmarks in the environment and thereby triangulate their current positions [12,13,15]. Additionally, they act as memory anchors for decision points, i.e. positions where the direction of travel needs to be adjusted [16].

In case of topographical maps empirical evidence exists showing that object location memory performance improves with increasing map complexity [17–19]. These authors also found that artificial map elements (grids) added to a map improve object location memory performance, especially in maps with low complexities. This counterintuitive finding may be best explained by the assumption that mental representations of space are generated based on spatial relations between single objects [20,21] that serve as reference points. Thus, the higher availability of reference points in complex topographic maps may support the formation of more accurate cognitive maps [1] up to some asymptote where the addition of detail no further improves memory performance (also see discussion in [19]). In case of route memory tasks, no such empirical data exists. Based on what we know from object location memory, we assume that adjusting a map display to increase visual complexity may also improve route memory. This question seems of particular interest in the context of volunteered geographic information (VGI) like OSM in the present study, where the amount of available detail depends on the engagement of their volunteers and the number of active VGI contributors in a specific area (e.g. [22]).

If web mapping services are used to plan a route, this route is displayed on top of the map layer. Based on the aspect ratio of the used device, a map scale is selected that allows to display the route in its entirety. Additionally, a margin is left around the route that prevents the route from intersecting the map borders. The map content displayed in this margin area contains spatial information, like landmarks, which act as navigation aids and can be used to generate a cognitive map [6,23,24]. Increasing this margin area would also increase the amount of spatial elements that can be displayed in the map, which may further improve the accuracy of the cognitive map. However, this would also decrease the size of the displayed route and, accordingly, its readability.

The tradeoff between displaying additional information around the route and ensuring a good visibility of the displayed route raises the question to what extent people use map elements such as landmarks offside a displayed route to memorize this route. If landmarks far offside the route are not used as spatial references, inserting large margins around the route would decrease readability of the route without any benefits. However, if landmarks offside the route play an important role in the formation of spatial representations, increasing margins around the route would be of advantage. In two consecutive experiments, we intended to identify factors affecting the task-relevance of landmark representations in route memory tasks based on their relative position to the route and the visual complexity of the map.

## Background

A precondition for the use of landmark representations as tools for route memory or navigation is their attentional processing. Whether map elements are processed is strongly affected by their salience, the tendency of an object to catch attention [25,26]. Concerning the attentional processing of landmark representations in route planning and navigation tasks, two subcategories of salience are expected to play an important role: visual salience and structural salience.

Visual salience defines the attention generated by physical characteristics of the landmark representations, like color contrast and the size of landmark pictograms [12,16]. Keil et al. [27] investigated the visual salience of OSM landmark pictograms using eye fixation measures and found large differences between the available pictograms. Without equalization of the visual salience of pictograms in web mapping services, effects of visual salience on the attentional processing of specific landmark representations cannot be avoided. However, in a controlled experiment the use of landmark pictograms with similar levels of visual salience is expected to reduce undesired effects on attentional processing.

**Fig 1:**
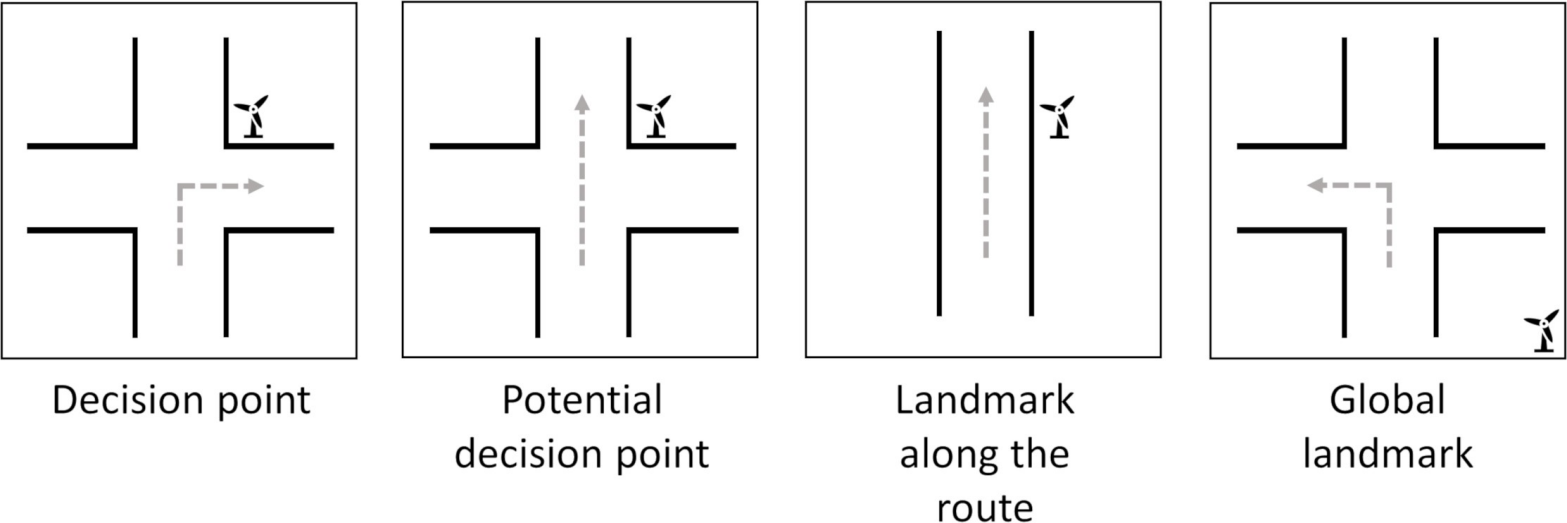
Possible positions of landmark pictograms relative to the route. Landmarks can be located close to decision points, potential decision points, along straight route sections or offside the route (adapted from Bauer [33]).

Structural salience on the other hand represents the degree of visual attention allocated towards an object based on its relative position to a specified route [16,28]. In navigation tasks, four types of route elements relevant for wayfinding instructions and route knowledge [13,29,30] can be distinguished (see Fig 1). *Decision points* are positions where at least two road branches exist (e.g. crossroads, T or Y junctions) and the route does not follow the previous course of the road. *Potential decision points* are positions with at least two road branches, but the route unambiguously follows the previous course of the road. Positions *along the route* are close to the route, but not close to any road branches. *Global* positions are offside the route and cannot be linked to specific route sections. Landmarks at *decision points* indicate that the direction of travel needs to be adjusted. Landmarks at *potential decision points* and along the route can be used to assure that the navigating person is still following the correct route. *Global landmarks* on the other hand are a special case. They are located offside the route and are only used to estimate cardinal directions [31,32]. Information about cardinal directions allows to identify an approximate travel direction, but it is not sufficient to follow an exact route. Therefore, we focus on the other three types of landmark positions in this study (at decision points, at potential decision points and along the route).

In addition, landmark representations can be located either close to the start or end of the route. As the start point of the route is the location of initial orientation, this location cannot be deduced from a previously identified location. However, once the start point has been identified in the map, people can apply counting strategies (e.g. take a right turn at the second crossroad) to update their current position and to support navigation and route memory [34]. As such strategies based on deducing the current position from a previous position are not functional for the start point of the route, a higher number of landmark pictograms acting as reference points could be required to identify this position than any other subsequent position along the route.

To understand which landmarks are required for memorizing a route and specific route segments, we need to assess which map elements are perceived and processed and which are not. In Geosciences and Geography eye tracking has been established as a measure to examine cognitive and attentional processing of map users [27,35–38] and to be able to examine different temporo-spatial strategies a user applies in route learning, navigation and other map reading tasks [39–42]. Of relevance for the examination of attentional processing of particular map objects are the fixation duration and the fixation count on these objects (defined as Areas-Of-Interest, AOIs, on the map). Both measures indicate attentional processing of spatial objects [35,43,44], as well as with the depth of cognitive processing of objects [45,46]. Therefore, in the present study these fixation measures will be used to examine attentional processing of landmark representations during navigation and route learning. Some first evidence now exists that the area distant from a to-be-learned route attracts less visual attention as indicated by fixation measures (see Keil et al. [47]), but the relation to map complexity and the processing of landmark representations has not been examined so far.

As we are interested in the attentional processing of landmark representations, we want to examine the likely relationship between visual map complexity, attentional processing and route memory. With regard to the structural salience of landmark representations, distances of landmark representations to decision points, potential decision points and the route in general will be investigated as potential predictors for their cognitive and attentional processing (as indicated by eye fixation measures). If structural salience directs visual attention towards landmark representations, we expect that the attentional processing of landmarks decreases with an increasing distance to the route, decision points and potential decision points of the route (H1). Second, landmark representations close to the start point of the route should receive especially high levels of attentional processing (H2). Third, route memory is expected to be better when the route is displayed in maps with high visual complexity (H3). Fourth, in maps with low visual complexity, map users are expected to use landmark representations as reference points that are further away from the route (H4). If we are able to verify the proposed relations between the visual complexity of maps, the relative position of landmark representations and their attentional processing in route memory tasks, we could deduce implications for map design. Findings could be used to select margin widths around displayed routes based on the requirement of reference points for memorizing a route.

## Experiment I

### Methods

The study was conducted in accordance with the Declaration of Helsinki. The used research design was controlled and approved by the ethics committee of the Faculty of Geosciences at the Ruhr-University Bochum (13 July 2018).

#### Participants

The study sample included 66 students of the Ruhr University Bochum (RUB). Exclusion criteria were neurological diseases or uncorrected poor eyesight. Based on quality criteria described in the statistics section, nine participants were removed from the final statistical analyses, which reduced the sample size to 57 participants (29 females, 28 males). The average age of the remaining sample was 22.8 (SD = 2.6), with a range between 19 and 30. Participants received a compensation of 5 EUR for participation in the study.

#### Materials

**Fig 2:**
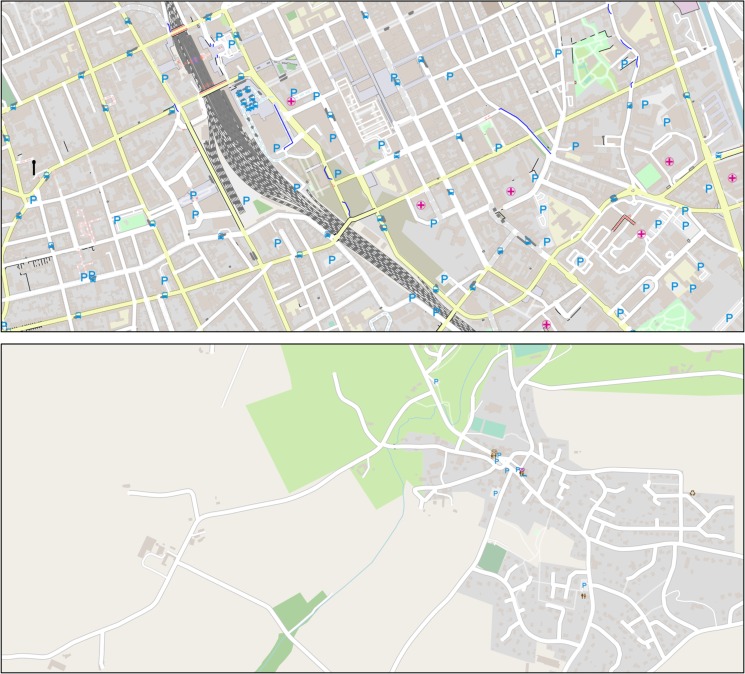
Map density conditions. The top half of the figure shows an example of an urban map (high visual complexity). The bottom half of the figure displays a map of a rural area (low visual complexity). The displayed maps were replicated with Maperitive using geodata obtained from OpenStreetMap.org.

Participants were randomly assigned to one of two between-subject conditions. Eight maps with a size of 45 x 20 cm were retrieved from OSM in a scale of 1:10,000. Four of these maps displayed urban areas with a high visual complexity, the other four displayed rural areas with a low visual complexity (***map density conditions***, see Fig 2).

**Fig 3:**
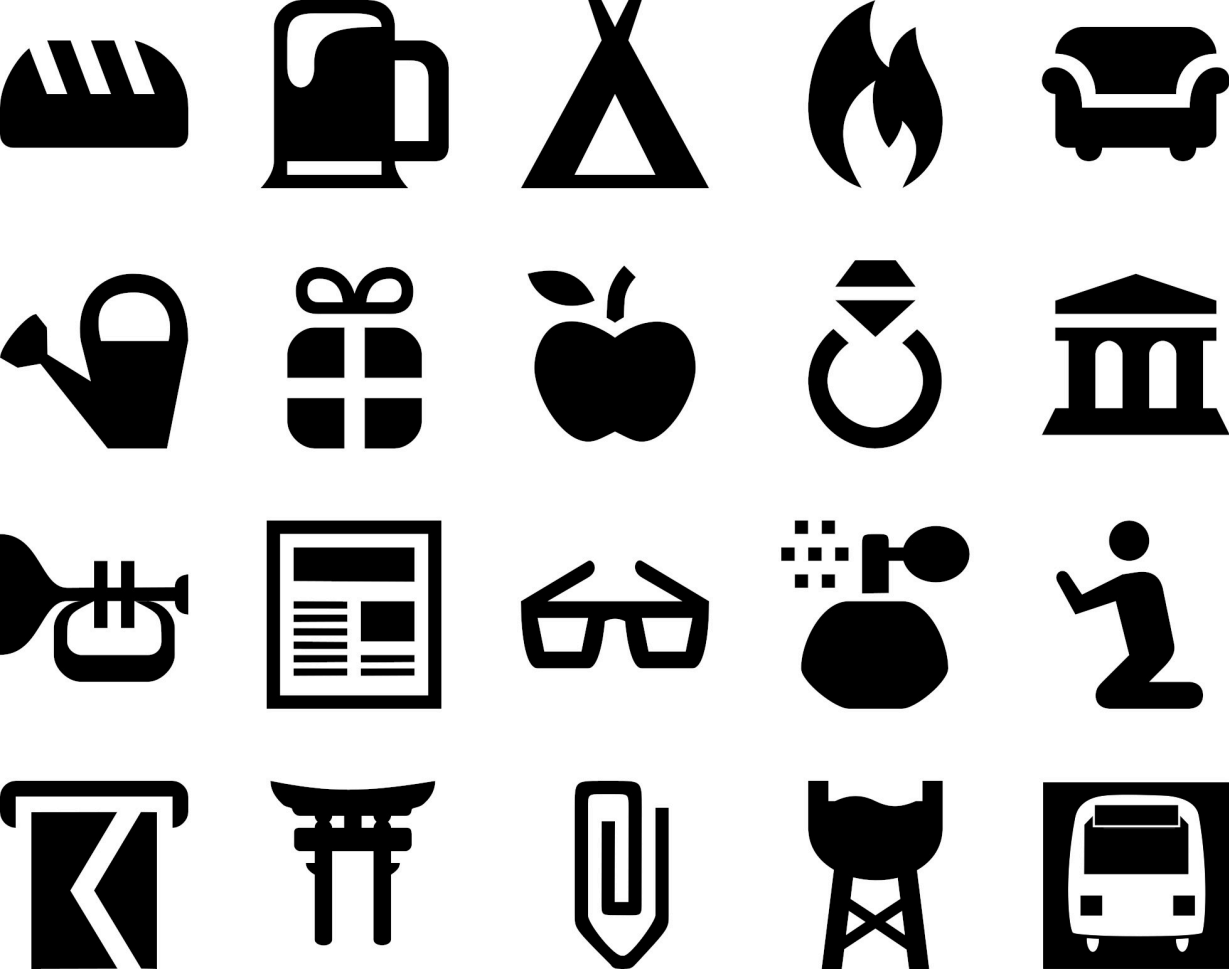
The used landmark pictograms. The displayed landmark pictograms were used as replacements for the original landmark pictograms in the stimulus maps. The selection of pictograms was based on findings of Keil et al. [27] with the aim to ensure similar levels of visual salience.

In order to validate our allocation of the selected areas as being either urban or rural, we compared the JPEG file size of the extracted maps. As JPEG file size correlates highly with subjective ratings of visual complexity [48,49], it can be used to differentiate between urban regions containing high amounts of map elements and rural regions containing only few map elements. Given that the mean file size (in KB) of our urban maps (M_Urban_ = 580.3, MIN_Urban_ = 479, MAX_Urban_ = 649) was higher than the mean file size of our rural maps (M_Rural_ = 191.8, MIN_Rural_ = 168, MAX_Rural_ = 219), we guaranteed a selection of representative map areas. After exporting all maps from OSM, a roughly horizontally running route containing six turnoffs (decision points) was drawn into each map (complete route). In order to control for potential effects of visual salience differences between landmark pictograms on visual perception, all landmark representations in the map were randomly replaced by a set of 20 OSM landmark pictograms (see Fig 3) assembled by Keil et al. [27], based on similar levels of visual salience.

**Fig 4:**
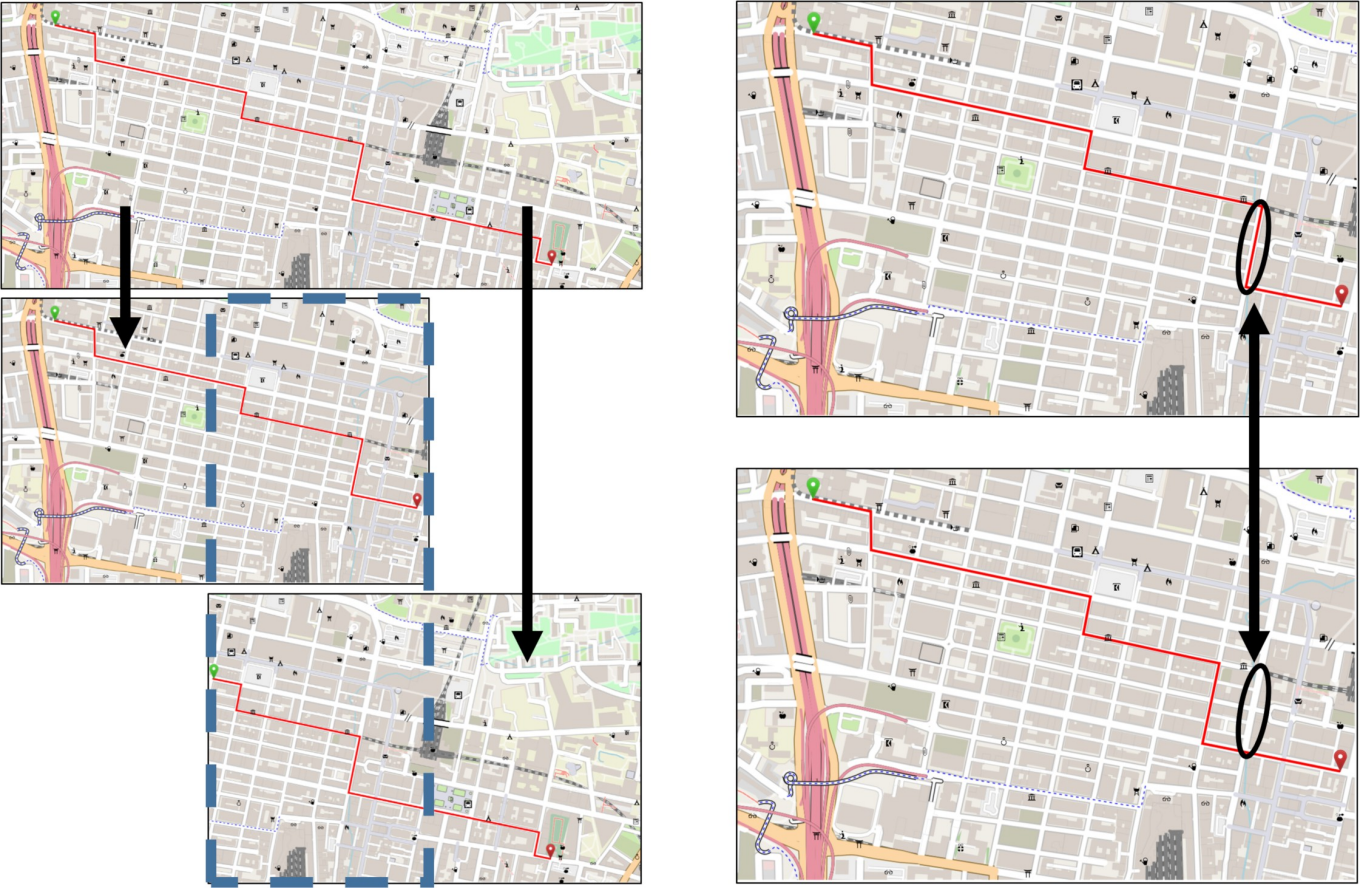
Stimulus design. The left side of the figure demonstrates how two study phase stimulus maps, with an overlap of 50%, were generated from a wide map (***map area conditions***). The overlapping area is highlighted by the blue dashed rectangle. The landmark pictograms in this area are, based on the condition, either close to the start or end of the route. The right side of the picture compares two recognition phase stimuli. The top map contains the correct route. The route in the bottom map is incorrect, as indicated by the black ellipses. The displayed maps were replicated with Maperitive using geodata obtained from OpenStreetMap.org.

Subsequently, two versions of each map were generated to be used as **study phase** stimuli. The first version displayed only the left two-thirds of the original map. The second version displayed only the right two-thirds of the original map (***map area conditions***). Accordingly, the two versions shared an overlap of 50% (see Fig 4). In each map, this overlapping area contained four of the six turnoffs of the complete route. As only two-thirds of the original map were used, either the start or end of the complete route was cut off. The routes in the study phase stimuli were shortened to prevent that they crossed the edge of the map.

For each of the 16 study phase stimuli (eight regions, two versions per region), four **recognition phase** stimuli were generated. These stimuli contained the same map as their corresponding study phase stimulus as well as a route. At least one of the four recognition phase stimuli showed the same route as the corresponding study phase stimulus. The other recognition phase stimuli contained a slightly modified route.

All study and recognition phase stimuli were exported as PNG files with a size of 30 x 20 cm (1133 x 755 pixels) and assigned to one of the two between-subject conditions (condition A or B) with an even distribution of left/right and urban/rural study phase stimuli.

#### Measures

*Attentional processing*. In order to assess attentional processing of landmark pictograms, we measured fixation durations and fixation counts using a Tobii TX-300 (300 Hz, 23 inches) eye-tracking monitor. Circular Areas-of-Interest (AOIs) were placed on each landmark pictogram, inside the overlapping area of the two map area conditions (the blue dashed rectangle in Fig 4). Using the eye-tracking software Tobii Studio (version 3.4.7), we calculated and exported the fixation counts and durations on each defined AOI per participant. Mean fixation durations were then calculated based on the fixation counts and total fixation durations. Concerning the diameter of the AOIs, it was important to consider an inherent tradeoff. As no eye-tracker has perfect accuracy, choosing a very small diameter would lead to many fixations on AOIs which are not recognized. In contrast, applying a large AOI diameter would score fixations close to a landmark pictogram as fixations on the pictogram. Therefore, we chose the AOI diameter by calculating a balanced proportion of AOI fixations being recognized according to the reported accuracy of the Tobii TX-300, which is 0.6° with a standard deviation of 0.7° for a gaze angle of 30° [50]. We selected a diameter of 60 pixels (1.58 cm). At a distance of 65 cm between the eyes and the eye-tracker monitor, this leads to a rate of on average 74.2% recognized fixations on AOIs.

*Relative landmark position*. The relative position of a landmark was measured based on its minimal distance to the route, to the next decision point of the route and to the next potential decision point of the route. The distance was measured in pixels. In accordance with the attentional processing measurement, only the landmarks in the overlapping map areas were investigated.

*Recognition performance*. Route recognition performance was assessed through a yes/no response task. In this task, participants were asked to compare routes with previously learned routes. Based on the signal detection theory (see 51), responses were scored as *hits* (correct route was recognized), *misses* (correct route was not recognized), *correct rejections* (route deviation was spotted) or *false alarms* (route deviation was not spotted). Hits, misses, correct rejections and false alarms were then translated into *d’* values, which represent the proportion of correct and incorrect responses [19,51,52] aggregated per participant to indicate recognition performance (i.e. how well participants are able to differentiate old from new items). d’ values above 0 indicate recognition memory performance above chance level.

#### Procedure

Preceding the start of the experiment, participants gave informed consent and the experimenter explained the procedure. As knowledge about the study purpose could have led to response biases [53,54], participants were told that a debriefing concerning the study purpose would take place after the experiment. After that, they were seated in front of the eye-tracker monitor with a distance of 65 cm between the eyes and the monitor. The study consisted of one training trial and eight experimental trials. At the beginning of each trial, participants were shown a stimulus map (study phase) for 30 seconds. Their between-subject condition defined whether the left or right version of a specific map (*map area conditions*) was shown. Both between-subject conditions contained the same amount of left and right map areas (four left/right map areas). Participants were required to memorize the route displayed in the map. After each study phase stimulus, the four corresponding recognition phase stimuli were displayed successively, each for eight seconds. After each recognition phase stimulus presentation, participants had to indicate whether the route displayed in the recognition phase stimulus matched the route in the corresponding study phase stimulus by pressing one of two keyboard buttons labeled with ‘yes’ and ‘no’.

#### Statistics

In order to test our hypotheses, we investigated the relations between the independent variables (visual complexity, map area conditions, relative landmark positions) and the recorded dependent variables (eye fixations, recognition performance). As mentioned above, the data of some tested participants was excluded from statistical analyses. Five participants had to be excluded, as the eye-tracker calibration was not successful. A second exclusion criterion was the completeness of the eye fixation data. Many factors as lighting, head movements or eye shape can affect the ratio of successful eye fixation recording [55–57]. If this ratio is low, important information concerning stimulus processing may be lost. As the remaining recorded eye gaze data may not be representative, including participants with low ratios of successful eye fixation ratios could lead to misinterpretations of their actual gaze patterns. Therefore, a minimum threshold of successful eye-tracking must be applied. Based on the suggestion of Bojko [58], we selected a threshold of 75%, which required us to remove the data of four additional participants from our analyses.

Our first hypothesis assumed relations between the distance of landmark representations to the route, decision points, potential decision points and the attentional processing of landmark representations. To test this assumption, eye fixation data was aggregated across participants to obtain one total fixation duration, mean fixation duration and fixation count value per landmark representation. Spearman correlations were then calculated between all fixation and distance measures.

Potential differences of the attentional processing of landmarks between the start point of the route and route sections near the end point of the route (H2) were examined by comparing the fixations on landmark representations between the two map area conditions using Spearman correlations.

Effects of visual complexity on route memory (H3) were assessed by aggregating recognition responses per map density condition to receive two d’ values per participant (one for urban maps and one for rural maps). Subsequently, d’ values were compared between urban and rural maps using the paired Wilcoxon signed-rank test.

Whether the visual complexity affected the distance of perceived landmark representations to the route, decision points and potential decision points (H4) was investigated by comparing the average of the mentioned distances between urban and rural maps. For this purpose, mean distance values were calculated per participant based on all landmark representations that were fixated at least once, but separately for urban and rural maps. Distance values were then compared between urban and rural maps with the paired Wilcoxon signed-rank test. Additionally, in order to test whether the distribution of landmark representations was similar in both map density (complexity) conditions, average distances of all landmark representations to the route, decision points and potential decision points were compared between urban and rural maps using Mann-Whitney U tests.

### Results

As shown in Table 1, all fixation measures (total fixation duration, fixation count and mean fixation duration) were highly negatively and significantly correlated to all three distance measures (distance to the route, distance to decision point, distance to potential decision point).

**Table 1:** Spearman correlations between fixations on landmark pictograms and their distance to the route, decision points and potential decision points. Values were aggregated across participants in order to create one value per landmark pictogram.

Variable	1	2	3	4	5
1. Total fixation duration					
2. Fixation count	.993***				
3. Mean fixation duration	.996***	.989***			
4. Distance to route	-.800***	-.804***	-.800***		
5. Distance to decision point	-.690***	-.692***	-.687***	.852***	
6. Distance to potential decision point	-.809***	-.811***	-.808***	.976***	.895***

* p < .003

** p < .0007

*** p < .00007, Bonferroni correction applied

All three fixation measures correlated positively and significantly when fixations on landmarks in the overlapping area were compared between the two map area conditions (see Table 2).

**Table 2:** Spearman correlations of fixations on landmark pictograms between the two map area conditions (landmark position close to the start or end of the route).

Variable	r_s_
1. Total fixation duration	.788***
2. Fixation count	.797***
3. Mean fixation duration	.788***

* p < .05

** p < .01

*** p < .001

The Wilcoxon signed-rank test showed no statistically significant difference of route recognition performance between the two map density conditions (M_Urban_ = 2.056, Mdn_Urban_ = 2.195, M_Rural_ = 2.192, Mdn_Rural_ = 2.199, W = 645, p = .15). The positive d’ values in both map density conditions demonstrate that the differentiation between correct and incorrect routes was above chance level.

**Fig 5:**
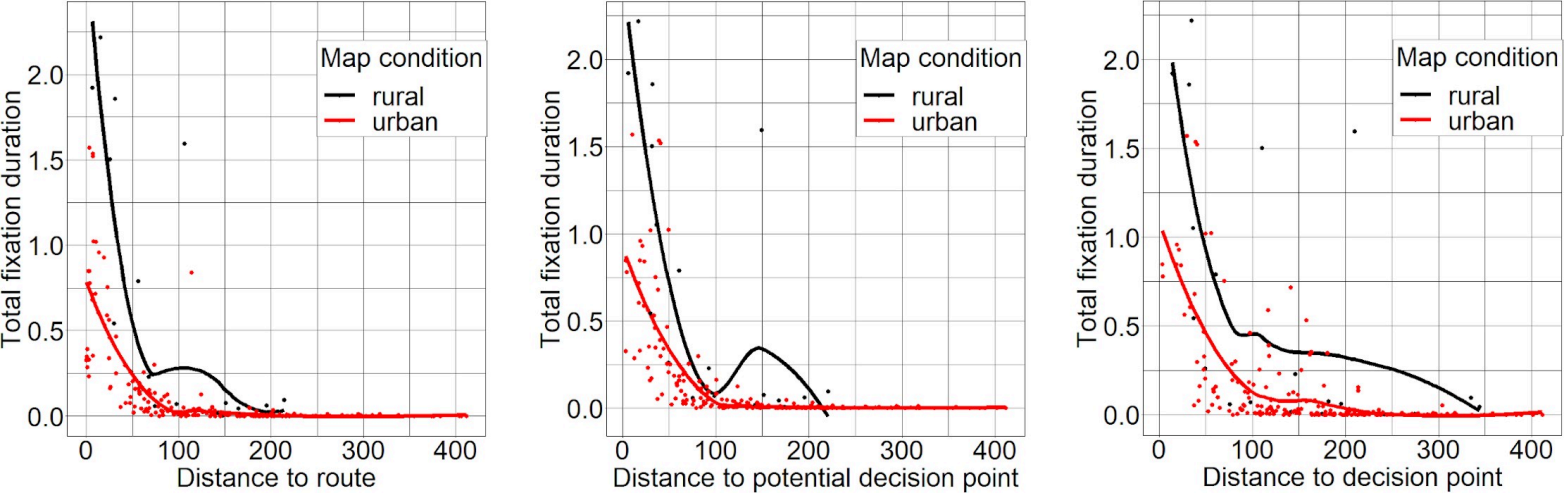
Relation between the total fixation duration on landmark representations and their distance to the route, decision points and potential decision points in pixels. The line graphs indicate that landmarks close to the route were fixated longer in the rural maps. They also show that urban maps contained more landmarks, especially far offside the route, decision points and potential decision points.

Although visual inspection of Fig 5 seems to indicate that participants looked at landmarks farther offside the route in the rural maps, statistical mean comparisons did not support this impression. The mean distance to the route of fixated landmarks (in pixels) did not differ significantly between the urban and rural maps (M_Urban_ = 41.52, Mdn_Urban_ = 39.53, M_Rural_ = 41.77, Mdn_Rural_ = 39.11, W = 831, p = .975). In contrast, the mean distance of fixated landmarks to decision points (M_Urban_ = 89.18, Mdn_Urban_ = 87.48, M_Rural_ = 67.88, Mdn_Rural_ = 59.94, W = 244, p < .001) and potential decision points (M_Urban_ = 51.45, Mdn_Urban_ = 49.33, M_Rural_ = 45.67, Mdn_Rural_ = 43.46, W = 539, p < .05) was even higher in urban maps. We also found that the average distance to the route (M_Urban_ = 142.15, Mdn_Urban_ = 125.22, M_Rural_ = 96.26, Mdn_Rural_ = 85.95, U = 1449, p = .063), decision points (M_Urban_ = 168.4, Mdn_Urban_ = 163.45, M_Rural_ = 139.59, Mdn_Rural_ = 126.17, U = 1556, p = .146) and potential decision points (M_Urban_ = 148.3, Mdn_Urban_ = 133.33, M_Rural_ = 107.22, Mdn_Rural_ = 95.52, U = 1480, p = .081) of all landmarks displayed in the maps was higher in urban maps. However, these differences were not statistically significant.

### Discussion

The negative correlations between the distance measures and the fixation measures (Table 1) demonstrate that, in line with our first hypothesis, landmark representations close to the displayed route, decision points and potential decision points were fixated more often. We conclude that these landmark representations have a higher structural salience. It may also be an indication that they are preferably used as reference points for memorizing the route.

Based on the high correlations of the fixation measures between the two map conditions we have to reject our second hypothesis. Participants fixated the same landmark representations independent of whether they were located close to the start or end point of the route. Therefore, increasing map margins close to the start point of the route does not seem to be important in future map design.

Inconsistent with our third hypothesis, no recognition memory performance differences were found between the two map density conditions. Thus, we cannot confirm that route memory performance is better in maps with high visual complexity. However, the lack of significant findings may have been caused by the low level of task difficulty. In fact, there were only very few incorrect responses in trials with both urban and rural maps. Additionally, the low visual complexity of rural maps may have affected the task difficulty in an undesired way. The rural maps did not only contain less landmark representations that could be used as reference points. They also contained less roads and less evenly distributed road structures. As Stevenage et al. [59] demonstrated that recognition performance is affected by the amount of distractors, using maps with unevenly distributed roads may have unwillingly led to a lower task difficulty, because mix-ups of roads were less likely and it was therefore easier to memorize what road sections were or were not part of the displayed route. This may be prevented by displaying comparable road layouts in routes with different levels of visual complexity. This limitation should be addressed in a follow-up study by using map stimuli with more similar road structures.

Given that the average distance of fixated landmark representations was not significantly higher in the rural maps, we cannot confirm our fourth hypothesis that maps with lower visual complexity motivate to adopt reference points farther offside the route for memorizing the route. We assume that the different distribution of landmarks across the map covered potential effects of map complexity on the attentional processing of landmarks offside the route. Although the higher mean distance of all landmarks to the route, decision points and potential decision points was not statistically significant, Fig 5 indicates that the urban maps contained many more landmark representations far offside the route than the rural maps. In order to overcome this limitation, we designed a second experiment. Experiment 2 was meant to replicate and extend the results of experiment 1 by using stimulus maps with different levels of visual complexity but a similar distribution of landmark representations across the map. While we expect to replicate the findings regarding the negative correlations between the distance of landmarks to the route and attentional processing, the second experiment was particularly designed to test the hypotheses of whether a lower visual complexity of a map leads to worse route memory and more attentional processing of landmark positions further away from the route.

## Experiment II

### Methods

In the second experiment, the same measures and the same procedure as in experiment 1 were applied. However, a new study sample and a new set of stimuli were used.

#### Participants

The study sample for the second experiment consists of 69 students of the Ruhr University Bochum. As in the first experiment, neurological diseases and uncorrected poor eyesight were exclusion criteria. Based on the quality criteria described in the previous statistics section, nine participants were removed from statistical analyses, leaving a sample size of 60 participants (28 females, 32 males). The age range of the remaining sample is between 18 and 32 (M = 23.9, SD = 2.8). Participants received a compensation of 5 EUR for participation in the study.

#### Materials

**Fig 6:**
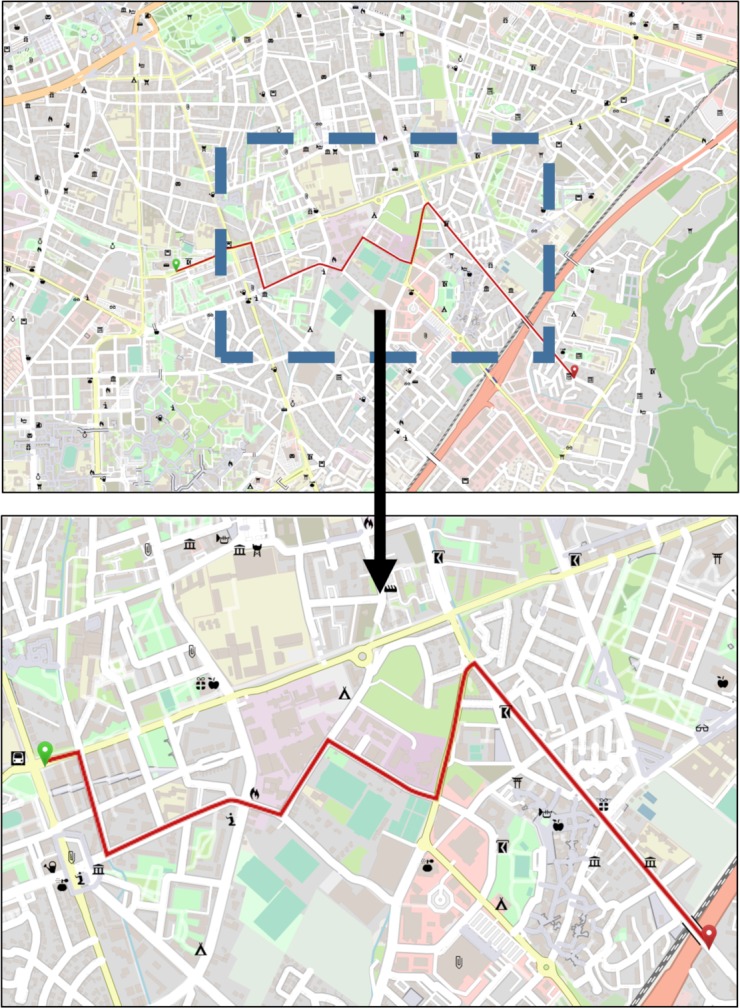
Stimulus design. The top map was retrieved from OSM in the scale of 1:12,500 and exported in the size of 30 x 20 cm (large region condition with a high visual complexity). The blue dashed rectangle (not visible in the stimulus) indicates the extraction area for the small region condition with a low visual complexity displayed in the bottom map (stretched from 13.5 x 9 cm to 30 x 20 cm). Therefore, the dashed rectangle also indicates the overlapping area between the two ***map area conditions***. The displayed maps were replicated with Maperitive using geodata obtained from OpenStreetMap.org.

As in the first experiment, participants were randomly assigned to one of two between-subject conditions. Eight maps with a size of 30 x 20 cm were retrieved from OSM in a scale of 1:12,500 (high visual complexity). A roughly horizontally running route with six turnoffs (decision points) was inserted into each map. Similar to the maps in the first experiment, each landmark representations in the map was replaced by a randomly selected OSM landmark pictogram from the set of 20 OSM landmark pictograms assembled by Keil et al. [27] based on similar levels of visual salience. Hereafter, a second variant (***map area condition***) was generated from each map by selecting a central map section with a size of 13.5 x 9 cm and stretching it to 30 x 20 cm (low visual complexity, see Fig 6). The route displayed in the stretched map was shortened to prevent it from crossing the map borders, but it still contained six turnoffs. Stretching the map area reduced the map complexity (elements per cm), while the relative distribution of landmark representations and the road structure remained similar between the two map area conditions. This was meant to overcome the likely bias induced by different task difficulties and landmark distributions of urban and rural maps in experiment 1. Stretching the map also increased the size of the landmark representations. However, as all map elements were increased by the same factor, visibility of landmark representations relative to other map elements did not change. Both the original sized and the stretched maps were used as **study phase** stimuli.

Similar to the first experiment, four **recognition phase** stimuli were generated for each of the 16 study phase maps. Again, these stimuli contained the same map as their corresponding study phase stimulus and either the same or a slightly modified route. At least one of the four recognition phase stimuli contained the same route as the study phase stimulus. All study and recognition phase stimuli were exported as PNG files with a size of 1133 x 755 pixels and assigned to one of the two between-subject conditions with an even distribution of non-stretched/stretched study phase stimuli.

#### Statistics

Matching the statistical analysis of the first experiment, the relation between the distance measures of landmark representations (distance to the route, decision points and potential decision points) and the attentional processing of landmark representations (H1) was assessed based on the mentioned distance measures and the fixation measures (total fixation duration, mean fixation duration and fixation count). After aggregating the eye fixation data across participants, Spearman correlations were calculated between the fixation and distance measures.

Inspired by the limitations found in the design of the first experiment, potential differences of route memory performance (H3) and landmark processing between maps with high and low visual complexity (H4) were not investigated by comparing urban and rural maps. Instead, route memory performance and landmark processing were compared between the original-sized and the stretched maps (map area conditions). This ensured a more similar road and landmark distribution between the two conditions. Recognition performance (d’) and distance values of landmarks to the route and (potential) decision points were aggregated across participants and map area conditions. Recognition performance was then compared between the map area conditions using the paired Wilcoxon signed-rank test. In order to compare the distance measures of fixated landmark representations between the map area conditions, independent samples Mann-Whitney U tests were applied.

### Results

Table 3 shows that all investigated fixation measures (total fixation duration, fixation count and mean fixation duration) were highly negatively and significantly correlated to all three distance measures (distance to the route, distance to decision point, distance to potential decision point).

**Table 3:** Spearman correlations between fixations on landmark pictograms and their distance to the route and (potential) decision points. Values were aggregated across participants in order to create one value per landmark pictogram.

Variable	1	2	3	4	5
1. Total fixation duration					
2. Fixation count	.993***				
3. Mean fixation duration	.991***	.981***			
4. Distance to route	-.882***	-.893***	-.877***		
5. Distance to decision point	-.756***	-.761***	-.738***	.771***	
6. Distance to potential decision point	-.867***	-.876***	-.858***	.965***	.810***

* p < .003

** p < .0007

*** p < .00007, Bonferroni correction applied

Concerning route recognition performance, no statistically significant difference of d’ values was found between the large map area condition with high visual complexity and the stretched area condition with low visual complexity (M_High_ = 0.953, Mdn_High_ = 1.095, M_Low_ = 0.982, Mdn_Low_ = 1.095, W = 456, p = .26). Similar to the results in the first experiment, both d’ values were positive. Hence, the ability to differentiate between correct and incorrect routes was above chance level in both map area conditions.

**Fig 7:**
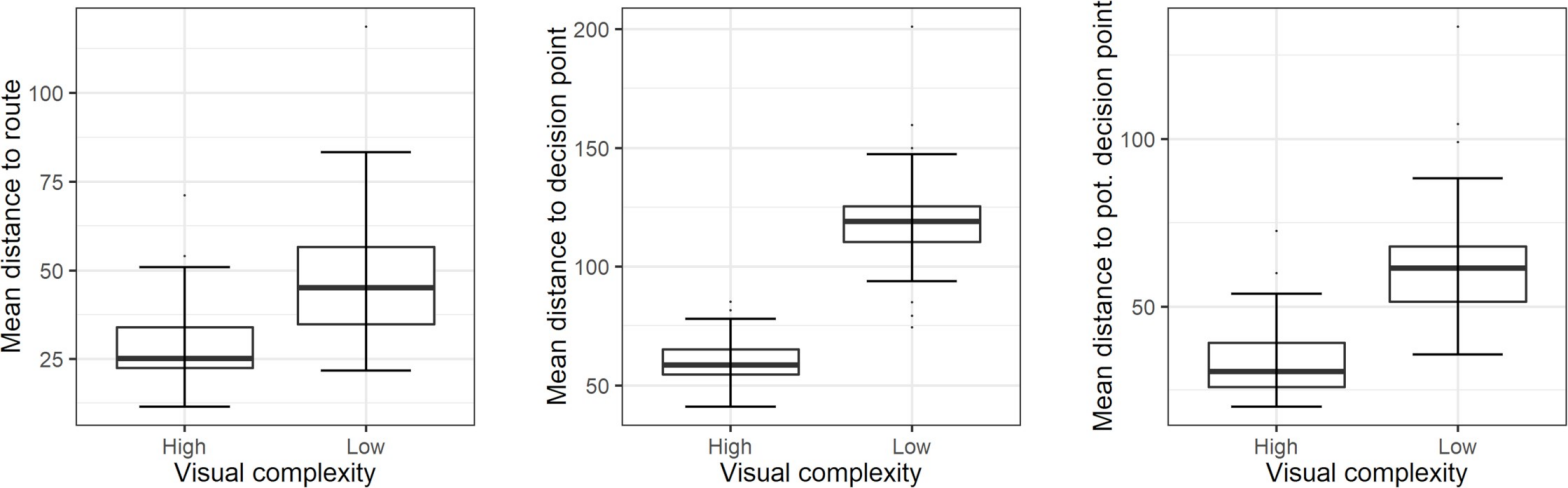
Mean distances of the fixated landmark representations per map area condition. The mean screen distance of the fixated landmarks to the route, decision points and potential decision points (in pixels) was significantly shorter in the large map areas with high visual complexity than in the stretched map areas with low visual complexity.

In contrast to the comparison between urban and rural maps in experiment 1, the mean distance (in pixels) of fixated landmarks to the route (M_High_ = 28.85, Mdn_High_ = 25.17, M_Low_ = 46.86, Mdn_Low_ = 45.07, U = 568, p < .001), decision points (M_High_ = 59.79, Mdn_High_ = 58.65, M_Low_ = 119.41, Mdn_Low_ = 119.15, U = 6, p < .001) and potential decision points (M_High_ = 33.95, Mdn_High_ = 30.54, M_Low_ = 62.69, Mdn_Low_ = 61.5, U = 173, p < .001) differed significantly between the two map area conditions (large area maps/stretched maps, see Fig 7).

### Discussion

Concerning the first hypothesis, the findings in the second experiment replicated the results of the first experiment. The closer landmark representations were to the route, a decision point or a potential decision point, the more often they were looked at.

Similar to the first experiment, route memory performance was not found to be affected by the visual complexity of a map. This contradicts the findings of Edler et al. [17] found for object location memory in topographic maps. Potential causes for the lack of significant differences of route memory performance between maps with varying visual complexity are presented in the general discussion.

Regarding our fourth hypothesis, using stimuli with a more similar distribution of landmark representations compared to the stimuli of our first experiment led to the confirmation of our prediction. In maps with lower visual complexity, and thus less reference points, people more frequently looked at landmark representations further offside the route and (potential) decision points. We have therefore found some evidence that people may require a certain amount of reference points to form spatial relations, and that they use more distant reference points if less reference points are available in close proximity to the route.

## General discussion and conclusion

The findings of the two described experiments enabled us to identify relevant factors for effective display of routes in maps.

Both experiments found clear indications for a strong negative relation between the visual perception of landmark representations and their distance to the route and (potential) decision points. The fact that similar results were obtained with different study samples and stimuli emphasizes the robustness of the findings. Hence, we can safely infer that the relevance of landmark representations for learning a route decreases with increasing distance to the route and (potential) decision points. This supports the assumption of Winter et al. [60] that the dominance of a landmark is inverse to its distance to an individual’s current position. It also fits to the findings of Keil et al. [47], which showed that areas offside a to-be learned route attract less visual attention. Although our results do not allow to deduce a definite recommendation for the width of map margins around a displayed route, they indicate that applying excessively wide margins is unlikely to improve route memory, especially, as this would simultaneously reduce the readability of the map and the displayed route. As the experiments were purely map based, it is important to mention that the pattern of attention towards specific landmark representations may differ if people have to perform real-world navigation tasks. In these cases, landmark visibility is likely to affect visual attention towards specific landmark representations. Thus, map representations of close landmarks that are hidden behind other objects are expected to attract less visual attention, whereas map representations of distant global landmarks are expected to attract more visual attention. Therefore, the findings may be generalized to map-based route planning, but not to real-world navigation tasks, as the relation between the visual perception of landmark representations and their distance to the route is expected to be much weaker. An additional question to be answered in future experiments is a potential interrelation of decision points and potential decision points concerning route memory performance. If people use close landmarks to memorize decision points, the presence of one or multiple potential decision points close to a decision point (and the memorized landmark) might lead to a mix-up between the decision point and a potential decision point. This could be investigated by manipulating the amount of potential decision points and their distance to the next decision point.

As demonstrated in the first experiment, perception of landmark representations close to the start point of the route is highly similar to the perception of landmarks further away from the start point of the route if they are placed at the same distance to the route and (potential) decision points. This implies that the distance to the route and (potential) decision points is more relevant than the distance to the start point of the route. Therefore, we see no necessity for increasing the amount of visible reference points around the start point of the route compared to other route sections, e.g. by increasing displayed margin regions around the start point of a route. Similar to the findings concerning the distance of landmarks to the route, it is important to consider how attention towards specific landmark representations may differ in real-world orientation tasks. For initial orientation, which was not required in the described experiments, people may use a mixture of visible local and global landmarks. Therefore, before the phase of planning and memorizing a route can be initialized, larger margin regions around the current location that also display global landmarks may be required.

Previous findings showing that visual complexity of a map increases memory performance in map-based memory tasks [17,18] were not replicated in the present experiments. Therefore, we cannot deduce recommendations for the size of applied map margin regions around a displayed route based on the visual complexity of the map region. One explanation for our lack of significant results could be that previous studies [17,18] used a location-based recall task instead of a route-based recognition task. Recall tasks usually have a higher level of difficulty [61,62], which promotes performance differences between experimental conditions. Therefore, applying a route recall task instead of a recognition task might uncover potential route memory performance differences based on map complexity. A second explanation could be that even though experiment 2 was intended to reduce the task difficulty differences between the two map area conditions in experiment 1, the low complexity map area might still have had an overall lower level of difficulty. Although the road structure was more similar than in experiment 1, the stretched low complexity map still contained less roads than the non-stretched map and therefore less possibilities for different route shapes. This might have compensated the assumed increased difficulty caused by the reduced amount of reference points in the stretched maps. In order to compare route recognition performance differences based on visual complexity differences, stimuli need to have even more similar road structures. Therefore, in follow-up experiments, we suggest to use the same map sections in both conditions and to modify the amount of all map elements excluding roads. Additionally, as learned from experiment 1, a similar distribution of map elements in both conditions should also be ensured. Still, comparing the different approaches it gets evident that the effect of visual complexity on recognition memory is clearly task-dependent. Finally, even if previous studies [17,18] found that location memory performance increased with map complexity, it cannot be deduced that the relation is linear. Other studies found that high visual complexity can distract from relevant stimuli, as more irrelevant stimuli are competing for visual attention [49,63]. Therefore, we assume that a tipping point exists where the benefit of having additional visual reference points usable for exact localization of objects is compensated by the difficulty to recover these reference points between competing visual stimuli. Thus, we assume that the relation between location memory performance and map complexity has an inverse u-shape (cf. [19]). Future experiments could investigate this assumption by investigating location memory performance in maps with extensively high visual complexity.

Our last hypothesis implied that eye fixation patterns in route memory tasks depend on the visual complexity of the used map. The first experiment found no statistical evidence for this hypothesis, which we argued to have been caused by an unequal distribution of landmarks across the stimulus maps, as landmarks in the maps with low visual complexity were on average closer to the route. However, the second experiment with a more similar distribution of landmarks found distinct differences of viewing patterns between maps with different levels of visual complexity. In maps with low visual complexity, people scanned a wider area around the route. These findings are in line with the assumption of Tversky [20] and McNamara & Valiquette [21] that people require reference points to form spatial relations as a foundation for a cognitive map. If less reference points are available in close proximity to the route, people seem to widen the scanned area in order to find suitable reference points for memorizing the route. However, our findings do not allow to explicitly ascribe correct route recall to the formation of a cognitive map. Even if people perceived landmarks and other spatial reference points, they may have memorized route shapes without relying on these reference points. To test whether people form a cognitive map based on spatial reference points and use it for memorizing the route, follow-up experiments should contain a control condition without spatial reference points. An additional aspect to consider in future experiments investigating effects of map complexity is the plausibility of the displayed map elements. In this study, findings from a previous study [27] were used to control for potential effects of visual salience on landmark fixation patterns. However, as different landmarks might be considered as unusual artifacts in rural or urban maps (e.g. a wind turbine in an urban area), plausibility of landmark pictograms in specific map areas might also affect fixation patterns. In order to prevent these potential effects on fixation patterns, landmarks that are plausible in rural as well as urban areas should be identified.

Based on our findings, we recommend to increase the margin regions around a displayed route with decreasing visual complexity of the region displayed in the map by either increasing the map size or decreasing the map scale. Follow-up experiments might investigate the implications for different map scale requirements (e.g. for pedestrians, cyclists or drivers) in the context of scale-driven map generalization (see Robinson [64]), or try to identify an ideal margin width around displayed routes based on the visual complexity of the map.

## Summary

The studies presented in this paper aimed to investigate how people use a map and map elements to memorize a displayed route. The results demonstrate that people primarily focus on the map area in close proximity to the route. The size of the surveyed area was found to depend on the visual complexity of the map. When a route was displayed in a map with low visual complexity, people looked at map elements (landmark representations) farther offside the route. This eye fixation pattern might be based on a requirement of spatial reference points for the formation of a mental representation of space. As the density of spatial reference points is lower in maps with low visual complexity, people need to scan wider areas in order to identify suitable spatial reference points. These findings can support task-oriented map design of web mapping services by coupling map scale or the size of displayed margin regions around a route to the visual complexity of the map.

## Supporting information

S1 Data (CSV)

S2 Data (CSV)
